# Silk‐in‐Silk Nerve Guidance Conduits Enhance Regeneration in a Rat Sciatic Nerve Injury Model

**DOI:** 10.1002/adhm.202203237

**Published:** 2023-02-25

**Authors:** Lorenz Semmler, Aida Naghilou, Flavia Millesi, Sonja Wolf, Anda Mann, Sarah Stadlmayr, Sascha Mero, Leon Ploszczanski, Lisa Greutter, Adelheid Woehrer, Eva Placheta‐Györi, Fritz Vollrath, Tamara Weiss, Christine Radtke

**Affiliations:** ^1^ Department of Plastic, Reconstructive, and Aesthetic Surgery Medical University of Vienna Spitalgasse 23 Vienna 1090 Austria; ^2^ Austrian Cluster for Tissue Regeneration Vienna 1200 Austria; ^3^ Institute of Physics and Materials Science University of Natural Resources and Life Sciences Gregor‐Medel‐Straße 33 Vienna 1180 Austria; ^4^ Department of Neurology Division of Neuropathology and Neurochemistry Medical University of Vienna Spitalgasse 23 Vienna 1090 Austria; ^5^ Department of Zoology University of Oxford Mansfield Rd. Oxford OX1 3SZ UK

**Keywords:** histomorphometry, mulberry silk, peripheral nerve injury, sciatic functional index, trichonephila silk

## Abstract

Advanced nerve guidance conduits can provide an off‐the‐shelf alternative to autografts for the rehabilitation of segmental peripheral nerve injuries. In this study, the excellent processing ability of silk fibroin and the outstanding cell adhesion quality of spider dragline silk are combined to generate a silk‐in‐silk conduit for nerve repair. Fibroin‐based silk conduits (SC) are characterized, and Schwann cells are seeded on the conduits and spider silk. Rat sciatic nerve (10 mm) defects are treated with an autograft (A), an empty SC, or a SC filled with longitudinally aligned spider silk fibers (SSC) for 14 weeks. Functional recovery, axonal re‐growth, and re‐myelination are assessed. The material characterizations determine a porous nature of the conduit. Schwann cells accept the conduit and spider silk as growth substrate. The in vivo results show a significantly faster functional regeneration of the A and SSC group compared to the SC group. In line with the functional results, the histomorphometrical analysis determines a comparable axon density of the A and SSC groups, which is significantly higher than the SC group. These findings demonstrate that the here introduced silk‐in‐silk nerve conduit achieves a similar regenerative performance as autografts largely due to the favorable guiding properties of spider dragline silk.

## Graphical abstract

Silk is emerging as versatile a biomaterial for regenerative medicine. Here, a silk‐based nerve conduit filled with spider silk fibers is used to treat a rat sciatic nerve defect. The silk‐in‐silk conduit achieves a similar regenerative performance as the current gold‐standard, the autograft, and represents a promising approach for nerve reconstruction.

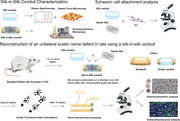

## 1 Introduction

Functional recovery of segmental peripheral nerve injuries remains a major challenge in restorative medicine. Despite continuous efforts, most patients suffer from lifelong disability, pain, and follow‐up surgeries.^[^ ^1^, ^2^ ^]^ Autologous nerve grafts derived from a sensory nerve are the current standard treatment.^[^ ^3^, ^4^ ^]^ These autografts provide endogenous structural support as well as pro‐regenerative cues and guidance from resident Schwann cells. However, the availability of donor nerves is limited, and their harvest creates an additional functional deficit and increases the risk for complications.^[^ ^5^ ^]^ A multitude of nerve guidance conduits composed of synthetic and natural materials have been developed as alternatives.^[^ ^6^, ^7^, ^8^, ^9^, ^10^, ^11^, ^12^, ^13^ ^]^ The currently available FDA‐approved nerve guidance conduits present hollow tubes whose application is restricted to short‐distance nerve defects of up to 3 cm.^[^ ^14^, ^15^ ^]^ Responsible for the inefficient nerve re‐growth over longer distances is the lack of an internal framework that provides structural and cellular support.^[^ ^15^, ^16^ ^]^ Without a scaffold and topographical cues that guide the ingrowth and alignment of Schwann cells, nerve regeneration is impeded by axon dispersion and failed re‐innervation. Hence, there is an ongoing search for suitable biomaterials and 3D scaffolds to construct next‐generation nerve conduits that emulate the nervous architecture and possess advanced biological and mechanical features supportive for regeneration.^[^ ^1^, ^17^, ^18^, ^19^, ^20^ ^]^

An increasing body of studies presents silk as an exceptional biomaterial with advantageous properties for the engineering of nervous tissue.^[^ ^21^, ^22^, ^23^, ^24^, ^25^, ^26^, ^27^, ^28^ ^]^ Silk of two arthropod classes, the insect *Bombyx mori* and the spider *Trichonephila species* (*Trichonephila sp*.), were studied in considerable detail.^[^ ^26^, ^29^, ^30^, ^31^, ^32^ ^]^ While spider dragline silk consists of the major ampullate proteins spidroin‐1, ‐2, and ‐3, silkworm cocoon silk is composed of fibroin and sericin proteins.^[^ ^23^, ^26^, ^33^, ^34^ ^]^ Sericin is associated with immunogenic properties, which requires its careful removal in a process referred to as degumming.^[^ ^29^, ^35^ ^]^ Sericin‐free silk fibers can be dissolved into a fibroin solution and reconstituted in silk‐only or multi‐material structures.^[^ ^24^, ^27^, ^36^, ^37^, ^38^ ^]^ The major advantages of fibroin solutions are the diverse processing methods that led to a variety of nerve conduits with different structural and mechanical features successfully applied in animal studies.^[^ ^11^, ^39^, ^40^, ^41^, ^42^ ^]^ In contrast, only insufficient spidroin solution can be generated for conduit fabrication due to the high effort/low yield of spider dragline silk harvest. The constantly improving production of recombinant spidroin, however, might provide a future alternative.^[^ ^43^, ^44^, ^45^, ^46^ ^]^

Spider dragline silk of *Trichonephila sp*. possesses remarkable mechanical properties combining high tensile strength and flexibility.^[^ ^47^, ^48^, ^49^ ^]^ Furthermore, dragline silk fibers are temperature‐stable from −75 to 230 °C, which allows for autoclaving for sterile application.^[^ ^30^, ^32^, ^50^ ^]^ In vitro studies demonstrated that native dragline silk served as a suitable substrate for the attachment and growth of Schwann cells, neuronal cells, and fibroblasts.^[^ ^51^, ^52^, ^53^, ^54^, ^55^ ^]^ When used in vivo, dragline silk showed long‐term degradability and hardly provoked any immune response.^[^ ^56^, ^57^ ^]^ Importantly, isogenic/decellularized veins filled with spider dragline silk resulted in a regenerative outcome similar to autografts after long‐distance nerve defects in rats and sheep.^[^ ^22^, ^58^, ^59^ ^]^ These results strongly encourage the favorable properties of spider dragline silk as internal guiding filaments for nerve conduits. However, the use of decellularized veins as conduit wall is dependent on donor tissue, costly, and only offers limited stability.

In this study, we tested an advanced nerve conduit that, for the first time, combines the features of commercial silk fibroin reconstituted into tubes and of natural spider silk filaments to generate a silk‐in‐silk conduit. The conduit wall was manufactured from *Bombyx mori* silk fibroin and filled with aligned *Trichonephila edulis* dragline silk fibers serving as an internal guiding structure. The material characteristics of the silk‐in‐silk conduit were analyzed and its regenerative performance was compared to empty conduits and autografts in a rat sciatic nerve injury model.

## 2 Results

### 2.1 High Resolution Microscopy Analysis

**Figure 1:**
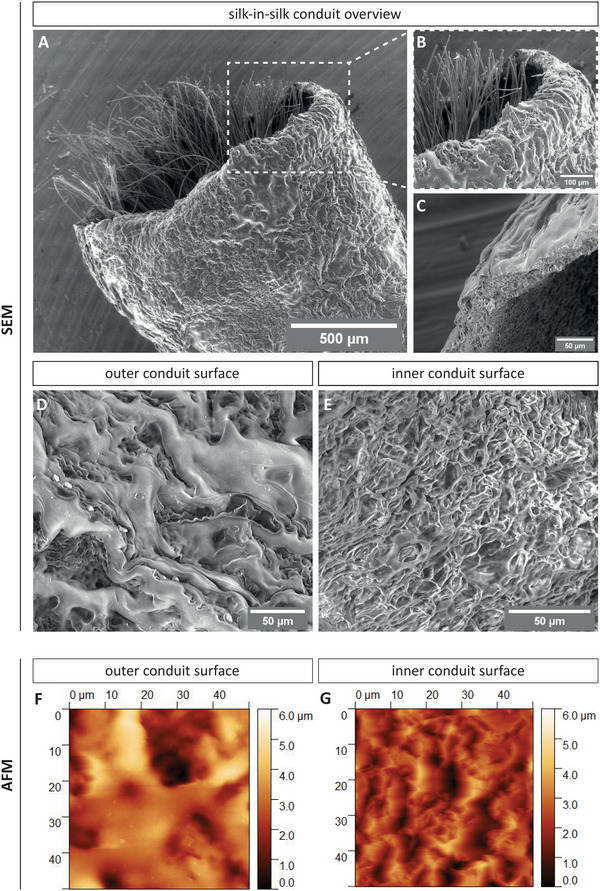
Scanning electron microscopy and atomic force microscopy analysis. Scanning electron microscopy (SEM) images. A) Representative micrograph of the silk fibroin tube containing longitudinally aligned spider dragline silk and B) magnification of the marked area. C) Micrograph of the silk fibroin conduit and magnification of the D) outer surface and E) inner surface displaying a different porosity. Representative atomic force microscopy (AFM) micrographs of the F) outer surface and G) inner surface of the silk fibroin conduit.

SEM and AFM analysis of the silk‐in‐silk conduits was performed to provide high resolution information about the surface topography. The SEM micrographs depict the silk fibroin conduit filled with dragline silk fibers (**Figure** **1**A,B) and the conduit itself (Figure 1C–E). In general, the conduit wall showed a high porosity, which is related to the treatment method during conduit production.^[^ ^39^ ^]^ Variations between the inner and outer conduit surface of the conduit wall can be seen in Figure 1C. The outer conduit surface possessed an inhomogeneous morphology with larger grains (Figure 1D), while the inner structure was characterized by rather uniformly distributed folds and pores (Figure 1E). The AFM micrographs confirmed the differences between the outer and inner conduit wall and determined a pore depth of single micrometers (Figure 1F,G).

### 2.2 Raman Spectroscopy

**Figure 2:**
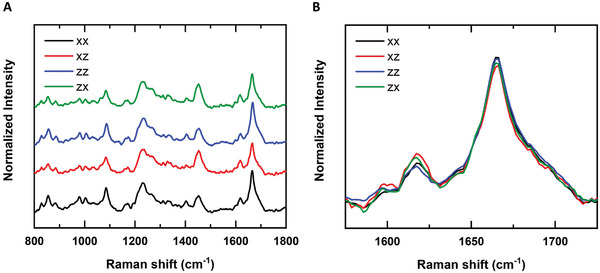
Raman spectroscopy analysis. A) Average polarized Raman spectra of the silk fibroin conduit. The amide I region and side chains (1575–1725 cm^−1^) are shown in detail in B). The spectra are normalized to the least square deviation of the *xx* spectrum.

Based on the Raman spectra the molecular composition of the silk fibroin conduit was identified (**Figure** **2**). The peaks ≈828, 856, 1174, and 1619 cm^−1^ could be an indication of the presence of Tyr, while the bands at 979 and 1084 cm^−1^ are attributed to Ser. The peaks ≈1004, 1403, and 1454 cm^−1^ can be assigned to Phe, Ala, and CH_3_ asymmetric bends, CH_2_ bending, respectively. In addition, the amide III (1200–1300 cm^−1^, centered at 1232 cm^−1^), and amide I (1600–1700 cm^−1^, centered at 1664 cm^−1^) are also clearly identifiable.^[^ ^60^, ^61^ ^]^ The spectral shape and center of both these regions is an indication of high content of *β*‐sheets.^[^ ^60^, ^61^ ^]^

The amide I region of the spectra shown in Figure 2A, were overlain on each other in Figure 2B to better allow the comparison of the intensities and deduce information about the orientation of proteins. As the amide I region is conformation sensitive, this band was further analyzed.^[^ ^53^, ^62^ ^]^ It is clear that the normalized intensities of the amide I band show similar intensities, which is especially the case for *xx* and *zz* measurements as well as *xz* and *zx*. This points out to a rather isotropic film that shows hardly any intensity changes with rotation in respect to the polarization of the incoming light. Indeed, these results are in in‐line with previous measurements on regenerated films from *Bombyx mori*, where no preferential alignment of the proteins, and by that no polarization effects during measurements were observed.^[^ ^63^ ^]^

### 2.3 Schwann Cell Attachment Analysis

**Figure 3:**
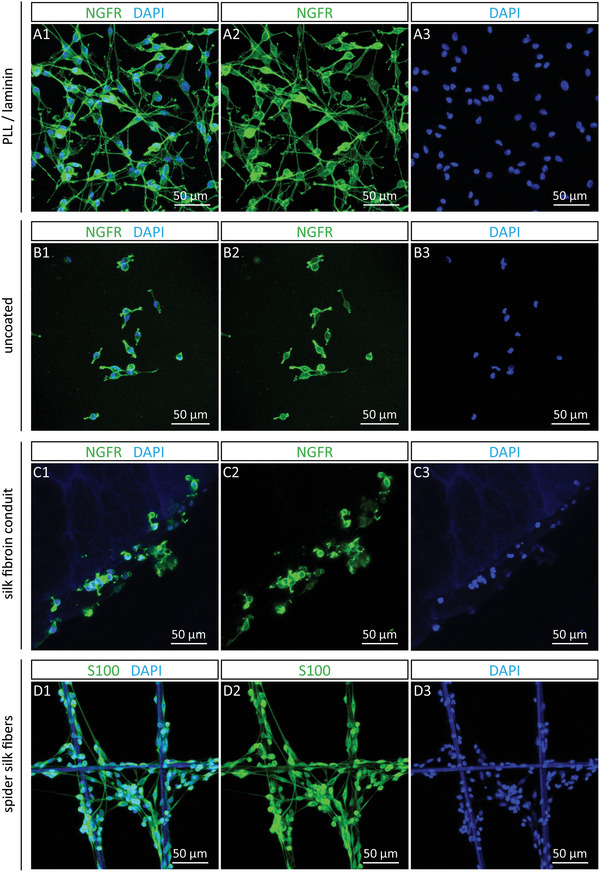
Immunostainings of rat Schwann cells grown on various substrates. Representative immunofluorescence images of rat Schwann cells grown on A) PLL/laminin coating, B) uncoated slides, C) the inner wall of the silk fibroin conduit, and D) spider silk fibers with merged channels (A1, B1, C1, D1), NGFR or S100 staining in green (A2, B2, C2, D2), and DAPI stain in blue (A4, B4, C4, D4).

As Schwann cells are essential to guide re‐growing axons across the injury site, we assessed cell attachment and morphology of rat Schwann cells seeded on 1) their ideal substrate, PLL/laminin coating, 2) uncoated plastic slides, 3) the inner wall of the silk fibroin wall, and 4) the dragline spider silk fibers. Schwann cell identity was confirmed by immunostainings for Schwann cell makers NGFR and S100 (**Figure** **3**). Schwann cells grown on the PLL/laminin coating showed their typical spindle shaped morphology with bipolar extensions (Figure 3A1–A3). The uncoated slides and silk fibroin wall allowed Schwann cells attachment, but the formation of extensions was reduced (Figure 3B1–B3,C1–C3). Of note, Schwann cells cultured on the spider silk fibers possessed a similar morphology to Schwann cells on the PLL/laminin coating (Figure 3D1–D3). These results illustrated that Schwann cells accept both the silk fibroin wall and spider silk fibers as growth substrates.

### 2.4 Functional Recovery

**Figure 4:**
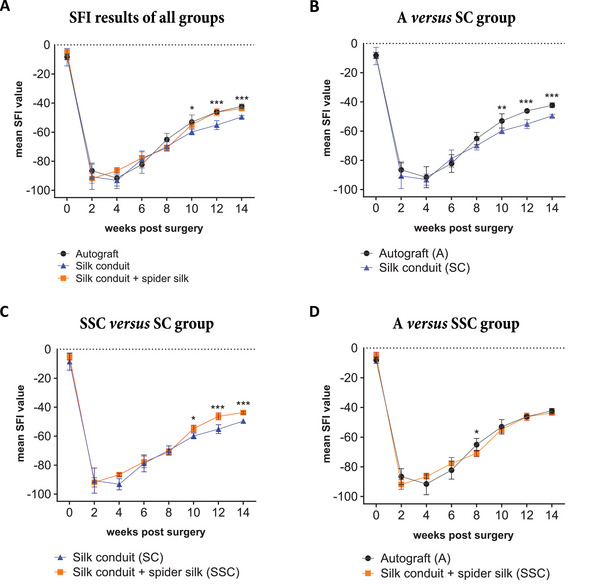
Comparison of SFI values between all groups until week 14 post surgery. A) Group wise comparison of the SFI results revealed a significant difference between the groups after 10 weeks post‐surgery. B) In the autograft (A) group versus the silk‐tube (SC) group, the mean SFI value was significantly increased in the A group from 10 weeks post‐surgery onward. C) In the silk‐in‐silk (SSC) group versus the SC group, the mean SFI value significantly raised in the SSC group from the ten‐week time point onward. D) In the A group versus the SSC group, a significant difference of the mean SFI value was only observed at the eight‐week time point. Values are depicted as mean ± SD. * = *p*<0.5; ** = *p*<0.01; *** = *p*<0.001.

To evaluate functional recovery of the animals, a gait analysis using the SFI pre‐operatively and every two weeks post‐surgery for 14 weeks was performed as described before.^[^ ^68^ ^]^ An SFI value of 0 indicates normal function, whereas negative results display impairment. All animals showed improved functional recovery from 4 weeks until 14 weeks post injury, but no group reached the preoperative values within the duration of the experiment (**Figure** **4**A). From week 10 onward, the statistical analysis of the SFI values showed a significant different group comparison (Figure 4A). The mean SFI values of both the A group (−53.01 ± 4.86) as well as the SSC group (−54.75 ± 2.32) were significantly increased compared to the SC group (−59.82 ± 1.99) (Figure 4B,C). Importantly, the A group and the SSC group demonstrated a similar functional recovery at weeks 10, 12, and 14 (Figure 4D).

### 2.5 Qualitative Evaluation of Nerve Regeneration

**Figure 5:**
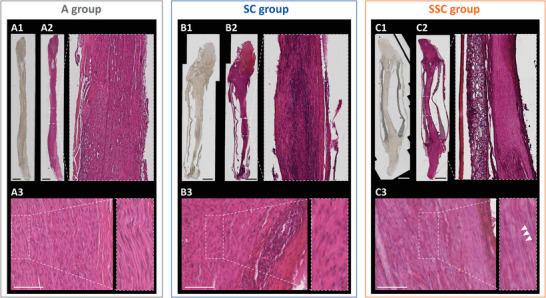
Qualitative evaluation of nerve regeneration in all groups 14 weeks post‐surgery. Representative sections of the whole regenerating nerve segment of the A) autograft (A) group, B) silk‐tube (SC) group, and C) silk‐in‐silk (SSC) group depicted as phase contrast images (A1, B1, C1) and after hematoxylin and eosin staining (A2, B2, C2; scale bars represent 1 mm) with enlargements of the middle part. Higher magnification images of respective sections are provided in A3, B3, C3, and Figure S2 (Supporting Information); arrowheads in C3 indicate one of many spider silk fibers that can be seen in the SSC group nerve section; scale bars represent 100 µm.

In order to display the regeneration status of the whole regenerated nerve segments, longitudinal nerve sections of all groups were microscopically evaluated using phase contrast microscopy (**Figure** **5**A1,B1,C1) followed by hematoxylin and eosin staining. Regrown nerve fibers were observed in all groups, but the regenerated tissue of the A and SSC group appeared more homogenous, while the tissue of the SC group contained dense conglomeration of cell nuclei (Figure 5A2,B2,C2). Representative enlargements illustrate dense intact nerve fibers in all groups (Figure 5A3,B3,C3; Figure S4, Supporting Information) with scant lympho‐monocytic infiltrates and blood remnants in the epineurium of the SC and SSC groups (Figure 5B3,C3). Of note, spider silk fibers can be identified as delicate filamentous structures in the SSC group (arrowheads in Figure 5C3; Figure S2, Supporting Information).

**Figure 6:**
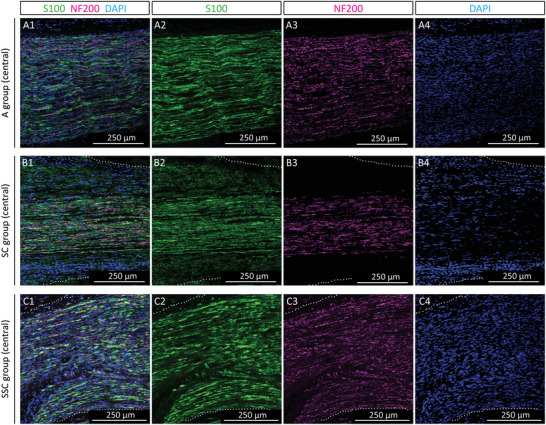
Immunostainings of longitudinal sections, central part, of all groups after 14 weeks post‐surgery. Representative immunofluorescence images of the central part of the A) autograft (A) group, B) silk‐tube (SC) group, and C) silk‐in‐silk (SSC) group with merged channels (A1, B1, C1), S100 staining in green (A2, B2, C2), neurofilament 200 (NF200) staining in magenta (A3, B3, C3), and DAPI stain in blue (A4, B4, C4). The white dotted lines indicate the silk conduit. In all groups, NF200 positive re‐growing axons are associated with S100 positive Schwann cells.

The regenerated nerve fibers were further analyzed by immunostainings for Schwann cell marker S100 and axon maturation marker neurofilament 200 (NF200). Images were taken at the proximal part (Figure S3, Supporting Information), central part (**Figure** **6**), and distal part (Figure S4, Supporting Information) and compared between the A, the SC, and SSC groups. S100 positive Schwann cells associated with NF200 positive axons were observed throughout the analyzed sections and confirmed ongoing nerve regeneration in all groups. In‐line with the hematoxylin staining, the SC group also contained conglomerations of S100 and NF200 negative cell nuclei, while equally distributed nerve fibers were observed in the autografts and the conduits filled with spider dragline silk (Figure 6B vs. C). Taken together, these results illustrate that the dragline silk fibers encourage a homogenous nerve re‐growth through the conduit.

### 2.6 Histomorphometry

**Figure 7:**
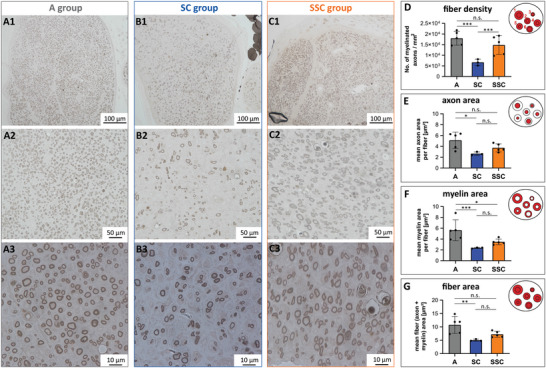
Histomorphometric evaluation of distal nerve segments after 14 weeks post‐surgery. Representative images and magnifications of osmium tetroxide stained myelin sheath on distal nerve cross‐sections of A) the autograft (A) group (n = 5), B) the silk‐tube (SC) group (n = 3), and C) the silk‐in‐silk (SSC) group (n = 5) used for semi‐automated image analysis. The bar diagrams in (D–G) depict the results for the A group in grey, the SC group in blue, and the SSC group in orange. D) The analyzed fiber density revealed higher numbers of myelinated axons / mm^2^ in both the A and SSC groups compared to the SC group. E) The mean axon area showed a significant difference between the A group and the SC group. F) Compared to the A group, the mean myelin area of both the SC group and the SSC group were significantly decreased. G) The myelinated fiber area (axon + myelin) was only significantly decreased between the A group and the SC group. Values are depicted as mean ± SD. * = *p*<0.5; ** = *p*<0.01; *** = *p*<0.001.

Myelinated axons were analyzed histomorphometrically in nerve cross‐sections distal to the autograft, the empty conduit, and the filled conduit. Representative images of distal nerve sections stained for myelin are illustrated for the A group (**Figure** **7**A1–A3), the SC group (Figure 7B1–B3), and SSC group (Figure 7C1–C3). Semi‐automated image analysis of these sections enabled to quantify the fiber density (number of myelinated axons/mm^2^), mean axon area (in µm^2^), mean myelin area (in µm^2^), and the mean fiber area (axon + myelin area in µm^2^). Notably, we detected a comparable nerve fiber density within the distal nerve segments of the A group (17984 ± 1444 mm^−2^) and the SCC group (14829 ± 1972 mm^−2^), while it was significantly decreased in the SC group (6646 ± 859 mm^−2^) (Figure 7D). Assessment of the mean axon area showed that there was no significant difference between the mean axon area of the A group (5.11 ± 0.68 µm^2^) and the SSC group (3.73 ± 0.33 µm^2^), but compared to the A group, the axon area was significantly lower in the SC group (2.3 ± 0.06 µm^2^) (Figure 7E). In addition to the axon content, also the myelination status of regenerated axons is a qualitative parameter for nerve regeneration. The largest mean myelin area was detected in the A group (5.63 ± 0.86 µm^2^) followed by the SSC group (3.48 ± 0.24 µm^2^) and was lowest in the SC group (2.36 ± 0.06 µm^2^) (Figure 7F). The quantified mean fiber area demonstrated no significant difference between the A group (10.74 ± 1.41 µm^2^) and the SSC group (7.21 ± 0.50 µm^2^), but was significantly reduced in the SC group (5.01 ± 0.21 µm^2^) (Figure 7G). These findings showed that the A and SSC groups had a comparable nerve fiber density and axon area, but myelination was most progressed in the A group.

## 3 Discussion

To address the need for off‐the shelf available nerve guidance conduits, our research focused on silk as it became of great interest for nervous tissue engineering due to its exceptional properties and versatile manufacturing possibilities.^[^ ^21^, ^24^, ^26^, ^36^ ^]^ The silk conduit wall used in this study was constructed from silk fibroin and processed into a porous structure.^[^ ^39^ ^]^ To promote a directed nerve regrowth, we enriched the fibroin conduit with aligned dragline spider silk fibers. This silk‐in‐silk conduit is the first to combine i) the excellent cell adhesion properties of native dragline spider silk filaments with ii) possible tissue regeneration advantages of fibroin‐silk based materials suggested by many bio‐medical studies.^[^ ^12^, ^16^, ^64^, ^65^, ^66^, ^67^ ^]^

First, we described the surface topography and molecular composition of the silk‐in‐silk conduit. SEM and AFM analysis confirmed the porosity of the constructed fibroin conduit, an essential feature for peripheral nerve guidance conduits by allowing nutrient and waste exchange.^[^ ^69^, ^70^ ^]^ Stability of nerve conduits is another essential aspect leading to their success in nerve regeneration by avoiding unfavorable effects, such as fractures and kinks. The silk proteins are well‐known for their high tensile strength.^[^ ^50^ ^]^ Our investigations of the molecular composition of the conduits with Raman spectroscopy demonstrated the presence of typical silk proteins with indications to a high content of *β*‐sheets.^[^ ^60^, ^71^ ^]^ This high content of *β*‐sheets has been shown to improve the mechanical properties of regenerated silk fibroin films^[^ ^72^ ^]^ and by that could be responsible for the conduit's stability. We further demonstrated that Schwann cells, the key drivers of peripheral nerve regeneration, accepted the fibroin conduit wall and dragline silk fibers as growth substrate.

The performance of the silk‐in‐silk conduit was then tested in a rat sciatic nerve injury model along with hollow silk conduits and autograft controls. Continual monitoring of the SFI served as functional read‐out and demonstrated a comparable recovery of rats treated with silk‐in‐silk conduits and the autografts. The qualitative evaluation of longitudinal nerve sections of the regenerated nerve segments by hematoxylin/eosin staining and immunostainings depicted a homogenous distribution of regenerating nerve fibers in the autograft and silk‐in‐silk conduit groups. In contrast, the silk conduit groups contained conglomerates of cells that could impair an organized nerve regrowth. In‐line with these results, the quantitative histomorphometric analysis determined a similar fiber density in the nerve segments distal to the silk‐in‐silk conduits and autograft controls, while significantly less fibers per mm^2^ were present in the segments distal to the empty conduit. Our findings support that the intraluminal dragline silk fibers had a beneficial effect on the number and distribution of re‐growing nerve fibers, as reported in previous studies. In a 20 mm rat sciatic nerve injury model, isogenic vein conduits filled with either Matrigel or longitudinally arranged *Trichonephila clavipes* dragline silk caused a significantly higher axon density in the spider silk group.^[^ ^22^ ^]^ Moreover, conduits composed of decellularized veins and intraluminal dragline silk fibers were used to treat long‐distance nerve defects in sheep and achieved a similar regenerative outcome as the autograft controls.^[^ ^58^, ^59^ ^]^ However, these studies utilized (processed) veins as conduits. With respect to a future clinical application, we here used a silk‐fibroin‐based conduit as off‐the‐shelf alternative. A previous study tested this fibroin conduit enriched with hyaluronic acid coated Spidrex fibers generated from degummed non‐mulberry silk fibroin.^[^ ^39^ ^]^ This conduit was applied to bridge a 10 mm gap of a rat sciatic nerve. After 12 weeks post‐surgery, the results demonstrated comparable outcomes in muscle endplate innervation and functional recovery between the autografts and conduits containing 200 Spidrex fibers.^[^ ^39^ ^]^ However, the regenerated fiber density and axon size within the distal nerve segment was significantly reduced in animals treated with the Spidrex conduits when compared to autograft controls. This is in contrast to our study, which showed a similar regeneration status of the nerve fiber density and axon area between the silk‐in‐silk and autograft groups. Hence, compared to Spidrex fibers, native spider dragline silk fibers possess characteristics advantageous for peripheral nerve regeneration. The increased regenerative effect of the silk‐in‐silk conduit on re‐growing axons is presumably caused by the superior interaction of cells with spider dragline silk. Accordingly, we confirmed that Schwann cells grown on the autoclaved dragline silk fibers showed long bipolar extensions. Of note, dragline silk does not require any further processing step, such as enzymatic treatment, coating, or modification with cell binding motifs to exert its favorable biological effect on cells.^[^ ^22^, ^51^, ^52^, ^53^, ^54^ ^]^ Importantly, native dragline silk was shown to provide an excellent adhesive surface allowing cell attachment, alignment, and migration for Schwann cells.^[^ ^52^, ^53^ ^]^ In response to injury, Schwann cells undergo transcriptional reprogramming to adapt a reparative phenotype accompanied by a profound morphological change.^[^ ^73^, ^74^, ^75^ ^]^ Denervated Schwann cells in the distal nerve segment extensively elongate and align within the basal lamina tubes to provide regeneration tracks, termed Büngner bands, for re‐growing axons.^[^ ^76^ ^]^ Native dragline silk was demonstrated to encourage this behavior by supporting the formation of sustained bundled structures of Schwann cells together with re‐growing axons along the silk in vitro.^[^ ^52^ ^]^ Moreover, the migration distance of Schwann cells seeded on dragline silk fibers achieved a remarkable speed of >1.1 mm per day, which is in‐line with the reported growth rate of naturally regenerating axons.^[^ ^4^, ^52^, ^77^ ^]^ These studies suggest that the rapid regeneration of axons through the silk‐in‐silk nerve conduit is based on the fast formation of Büngner band like structures along the luminal dragline silk fibers.

Indeed, the silk‐in‐silk conduit achieved similar results to studies using acellular nerve grafts, collagen conduits with intraluminal fillers and autologous cells with comparable nerve defects, regeneration time, and read out.^[^ ^78^, ^79^ ^]^ However, the silk‐in‐silk conduit allows to introduce additional modifications to further boost its regenerative performance. One major benefit of fibroin‐based conduits are the versatile incorporation possibilities of bioactive molecules during the production process. Previous studies demonstrated that silk fibroin conduits enriched with growth factors, either applied via silk microspheres or entrapped in the conduit wall by cross‐linking or adsorption, had a positive effect on nerve regeneration in rat sciatic nerve defect models.^[^ ^40^, ^42^, ^80^ ^]^ Hence, improvement of the herein presented silk‐in‐silk conduit by loading the conduit wall with growth factors, matricellular proteins or other molecules could enhance Büngner band formation and nerve fiber re‐growth. In addition, the silk‐in‐silk conduit may be enriched with biofunctionalized silk fibroin hydrogels and cells as additional source for neurogenic support. Thus, the silk‐in‐silk conduit encourages further enhancement into a multifunctional nerve guidance conduit as therapeutic alternative for patients suffering from long‐distance segmental nerve injuries.

## 4 Conclusion

This work introduces a pure silk nerve guidance conduit as advanced approach to encourage peripheral nerve regeneration across gap injuries. We report a comparable regenerative performance of animals treated with autografts and the silk‐in‐silk nerve conduits. With regard to clinical translation, experiments with larger gap sizes and inclusion of upper extremity nerve injury models are necessary. To this end, the silk‐in‐silk conduit allows biofunctionalization of the conduit wall and the introduction of hydrogels to meet the needs of critical segmental nerve defects.

## 5 Experimental Section

### Spider Silk Harvest

Female golden orb‐web spiders *Trichonephila edulis* were housed as described previously.^[^ ^53^ ^]^ For the dragline silk harvest, the spider was fixated under a gauze and the dragline silk was carefully derived from the major ampullate gland.^[^ ^54^ ^]^ The silk was reeled on metal frames and autoclaved (Vacuklav, 43‐B) at 121 °C and 1.1 bar. These conditions were held for 20 min and 30 s.

### Silk‐in‐Silk Conduit Preparation

The conduit wall was constructed from a concentrated 8–10% solution of dialyzed regenerated *Bombyx mori* silk fibroin produced by Oxford Biomaterials Ltd, UK (OBM).^[^ ^81^, ^82^, ^83^ ^]^ The autoclaved dragline spider silk was inserted into the fibroin conduits before implantation under sterile conditions. One spider silk harvest resulted in ≈10 m of silk, suitable to fill one 10 mm long fibroin conduit.

### Scanning Electron Microscopy

Scanning electron microscopy (SEM) of the prepared silk‐in‐silk conduits was performed with a Quanta 250 FEG, FEI device (ThermoFisher Scientific) by means of a secondary electron detector. Micrographs were obtained in low vacuum (100 Pa), to allow imaging without the need of a conductive layer. The stage was tilted orthogonally to the detector for the access to the inner side of the conduits.

### Atomic Force Microscopy

For atomic force microscopy (AFM) imaging, pieces of the silk conduit were cut with a Stanley knife, carefully flattened with a forceps, and fixed on glass microscope slides with double‐sided tape. A multimode atomic force microscope (Ntegra Aura, NT‐MDT) was used. Randomly chosen positions of the conduit were imaged under ambient conditions in the tapping mode. Standard tapping mode cantilevers (NSG30, NT‐MDT) with a force constant of ≈40 N m^−1^ were employed. Following the imaging, the AFM micrographs were post‐processed using the software Gwyddion (GNU General Public License). Corrections of the background were performed via a polynomial function, rows were aligned using the median of differences and artefacts were removed with the functions “stepline correction” and “remove scars”.

### Raman Spectroscopy

The molecular structure of the conduit was investigated by Raman spectroscopy. A WITec alpha 300A micro‐Raman device and a 10x/0.23 objective were employed. A frequency doubled Nd:YAG laser at a wavelength of 532 nm and an average power of 30 mW were used. The spectrometer has a resolution of 2 cm^−1^ and the experiments were performed in a backscattering geometry. Each measurement was performed for 100 s and no change of the sample morphology was observed afterward. Three points on the conduit were randomly chosen and measured.

The proteins of the cocoon fibers from *Bombyx mori* in their natural form show an orientation, where the molecular chains were aligned parallel to the axis of the fiber.^[^ ^62^, ^71^ ^]^ Hence, in order to investigate the changes in the protein orientation during the film preparation, four polarized spectra per spot were taken: the polarization of the incoming laser light was oriented either parallel (*z*) or perpendicular (*x*) to the long axes of conduit and a polarizer before the detector allowed detection of signal also in *z* and *x* directions. Hence, measurements in *xx*, *xz*, *zx*, and *zz* orientations were possible.^[^ ^60^ ^]^ The spectra were smoothed, baseline corrected with a polynomial function, and normalized to the least square deviation of the *xx* spectrum. This allows a direct comparison of the peak intensities and drawing conclusions about whether the proteins in the conduit were oriented or isotropic.

### Schwann Cell Culture

Rat Schwann cells were isolated, cultured, and purified as described previously.^[^ ^52^, ^84^ ^]^ Schwann cells were thawed and cultured on 0.01% poly‐l‐lysine hydrobromide (PLL, Sigma–Aldrich) and 4.8 µg mL^−1^ laminin (Sigma–Aldrich) coated dishes in culture medium consisting of MEM ∝ (GIBCO) supplemented with 1% Penicillin–Streptomycin (GIBCO), 1% Sodium Pyruvate Solution (GIBCO), 2.5% HEPES (GIBCO), 0.5% N‐2 Supplement (GIBCO), 2 µM forskolin (Sigma–Aldrich), 10 ng mL^−1^ recombinant Heregulin*β*‐1 (PeproTech), 10 ng mL^−1^ recombinant FGF‐basic (PeproTech), 5 ng mL^−1^ PDGF‐AA (PeproTech), and 5% fetal calf serum (LINARIS). Half of the medium was changed three times a week.

In vitro experiments: 5 × 10^4^ rat Schwann cells were seeded per well of a PLL/laminin coated or uncoated 8‐well chambered microscopy slide (Ibidi) or on the inner wall of a longitudinally cut silk fibroin conduit and cultured for 5 days before immunostaining. Rat Schwann cells (15 × 10^4^) were seeded on dragline spider silk reeled on metal frames that fit in a well of a 2‐well chambered microscopy slide (Ibidi)^[^ ^52^, ^53^ ^]^ and cultured for 2 weeks before immunostaining. For the assessment of cell attachment and morphology, Schwann cell cultures derived from a minimum of four different donors were analyzed.

### Immunofluorescence Staining of Grown Schwann Cells

Cells were washed twice with 1xPBS for 5 min each and fixed with 4.5% formaldehyde solution (SAV Liquid Production GmbH) for 20 min at room temperature (RT). Blocking and permeabilization was performed with 1xPBS containing 1% BSA, 0.3% TritonX‐100 (Sigma–Aldrich), and 5% goat serum (DAKO) for 10 min at RT. The cells were then incubated with the primary antibodies (NGFR, rabbit, 1:400, CellSignaling, #8238S; or S100, rabbit, 1:300, DAKO, #Z0311) in 1xPBS containing 1% BSA, 0.1% TritonX‐100 (Sigma–Aldrich), and 1% goat serum overnight at 4 °C. The next day, cells were washed three times with 1xPBS and incubated with the secondary antibody anti‐rabbit AF488P (1:800, Invitrogen, #A32731) for 1 h at RT. For DNA staining, 50 µg mL^−1^ 4′,6‐Diamidino‐2‐Phenylindole (DAPI, ThermoFisher Scientific) was added to the cells for 10 min. After washing, cells were mounted with FluoromountG (ThermoFisher Scientific). Pictures of stained cells were taken using a laser scanning confocal microscope (Leica SP8X) with a 20x multi‐immersion objective (Leica) and 0.75 or 1.5 zoom and are depicted as maximum projection of total *z*‐stacks.

### Animal Model and Surgical Procedure

Three groups of 12 weeks old male Sprague Dawley rats (n = 6/group) between 300–350 g were used in this study. Rats were anesthetized with 100 mg kg^−1^ ketamin (Livisto) and 5 mg kg^−1^ xylazin (Bayer), and intubated orotracheal with 40% O_2_ and 2% Isofluran (Zoetis). The right sciatic nerve was exposed from a transgluteal access and a defect was created by cutting out a 10 mm nerve piece using microsurgical equipment. The nerve defect was treated with a reversed coapted autograft (A), a hollow silk fibroin conduit (SC), or a silk fibroin conduit filled with spider dragline silk fibers (SSC). The autologous nerve graft as well as the conduits were coapted with three epineurial 10–0 Ethilon sutures.

All animal experiments were carried out in accordance with the applicable international, national, and institutional guidelines for the care and use of animals. Animal studies were approved by the Federal Ministry for Science and Research (BMWF), Vienna, Austria (BMWFW‐66.009/0259WF/v/3b/2017).

### Functional Analysis

Walking track analysis was performed pre‐operatively and every 2 weeks post‐surgery for 14 weeks to evaluate functional outcome of nerve regeneration.^[^ ^85^ ^]^ Briefly, footprints of the injured and uninjured hindlimbs were analyzed for print length, toe spread, the distance between the first and fifth toes, the intermediate toe spread, and the distance between the second and fourth toes. The Sciatic Functional Index (SFI) was calculated according to Bain et al.^[^ ^68^ ^]^

### Processing of Nerve Tissue/Nerve Harvest

After completion of the functional analysis 14 weeks post‐surgery, rats were euthanized by intraperitoneal injection of 600 mg kg^−1^ sodium pentobarbital. Segments of the sciatic nerves (2.5 cm) including the conduits or autografts and the distal nerve region were harvested for further processing. An overview of the silk conduit and the silk‐in‐silk conduit before and after implantation as well as 14 weeks post‐surgery (in vivo and ex vivo) could be found in Figure S1 (Supporting Information).

The excised nerve segments including the conduits or autografts were fixed in 4.5% formaldehyde solution for 48 h, dehydrated, and embedded in paraffin using a KOS Microwave HistoStation (Milestone). The conduit or autograft containing segments were cut in 8 µm thick longitudinal sections using a microtome (Leica RM 22359). Phase contrast images of whole nerve sections of the A, SC, and SSC group were taken with a benchtop microscope (NIKON Eclipse Ts2R) followed by hematoxylin/eosin staining and immunostainings. Cross‐sections of the distal nerve end of the segments were prepared for histomorphometric analysis.

### Hematoxylin and Eosin Staining of Nerve Sections

Conventional hematoxylin and eosin stainings of whole nerve sections were performed to visualize morphological features of cell nuclei (purplish blue staining) as well as cytoplasmic and extracellular compartments (shades of pink) of regenerating nerves. Briefly, the nerve sections were deparaffinized, hydrated with decreasing ethanol series (100%, 96%, 80%, 70%, and 50%) and washed in distilled water. Slides were immersed with hematoxylin (Merck) for 5 min and rinsed under running tap water for 10 min. Samples were then put in eosin (Sigma–Aldrich) staining solution for 1 min, briefly washed with distilled water and dehydrated with increasing ethanol series (70%, 96%, and 100%). Sections were finally cleared in *n*‐butylacetate and mounted with entellan. Images were taken with a benchtop microscope (NIKON Eclipse Ts2R) or a NanoZoomer Slide Scanner (Hamamatsu).

### Immunofluorescence Analysis of Nerve Sections

The longitudinal nerve sections were stained for S100 (rabbit, 1:200, DAKO, #Z0311) and neurofilament‐200 (chicken, 1:300, ThermoFisher Scientific, #PA1‐10002). The sections were incubated with the primary antibodies for 2 h at RT, washed with 1xPBS, and then incubated with the secondary antibodies anti‐rabbit AF488P and anti‐chicken DL650 (1:400, ThermoFisher Scientific, #SA5‐10073) for 1 h at RT. Then, 50 µg ml^−1^ DAPI was added for 10 min. After washing, the sections were mounted with FluoromountG, covered with a coverslip (VWR) and sealed with glue (Marabu). Images were acquired with a confocal laser scanning microscope (Leica, SP8X) and were depicted as maximum projection of total *z*‐stacks. Contrast and brightness had been adapted in a homogenous manner and pseudo coloring was applied to make multicolor figures comprehensible for color‐blind readers.

### Histomorphometric Evaluation

Nerve segments distal to the conduit or autograft were processed for histomorphometric analysis according to established histological stainings.^[^ ^86^, ^87^, ^88^ ^]^ Briefly, the tissue was fixed in 3% glutaraldehyde (Sigma–Aldrich) for 24 h and stored in 0.1 mol l^−1^ cacodylate at 4 °C until further processing. The tissue was postfixed with 2% osmium tetroxide (Electron Microscopy Sciences), a strong oxidant that reacts with unsaturated double bonds, which results in the deposition of osmium black and the staining of myelin. After embedding in epoxy resin, 1 µm cross‐sections were cut using an ultra‐microtome (Leica Ultracut UCT). The sections were incubated with 1% para‐phenylendiamin (PPD, Sigma–Aldrich), which additionally stains the myelin sheath, and examined by light microscopy (Nikon Eclipse Ni). Histomorphometric measurements of these sections were performed with a semiautomatic image‐analyzing system (LUCIA‐M, Nikon Laboratory Imaging). From the identified myelinated fibers, the myelinated fiber density (number of myelinated axons per mm^2^), the mean axon area in µm^2^, the mean myelin area in µm^2^, and the mean myelinated fiber area (axon + myelin area) in µm^2^ were calculated.

### Statistical Analysis

All data are reported as the mean ± standard deviation. One‐way ANOVA and Tukey Post‐Hoc‐Analysis were performed using SPSS Statistics 25 (IBM). Graphs were created with GraphPad Prism6 software. A *p*‐value of <0.05 was considered as statistically significant.

## Conflict of Interest

F.V. has equity interests in two Oxford University Spin‐offs, i.e., Oxford Biomaterials Ltd (which provided the silk tubes for this research) and Newurotex Ltd (which is developing a silk‐based device for peripheral nerve repair).

## Author Contributions

T.W. and C.R. contributed equally to this work. Conceptualization: L.S., E.P.G., C.R. Methodology & Investigation: L.S., A.N., F.M., S.S., T.W., A.M., L.G., S.M., S.W., C.R. L. Material: L.S., F.M., S.S., F.V. Software: L.S., T.W., S.W., A.N., S.S. Visualization: T.W., L.S., A.N., F.M. Formal Analysis: L.S., T.W., A.N., A.W. Writing & Draft Preparation: T.W., A.N., L.S. Writing‐review & Editing: all authors; Supervision: T.W., A.N., C.R. Funding Acquisition: C.R.

## Supporting information

Supporting Information

## Data Availability

The data that support the findings of this study are available from the corresponding author upon reasonable request.
