# Supplementary material for: Silk‐in‐Silk Nerve Guidance Conduits Enhance Regeneration in a Rat Sciatic Nerve Injury Model
Source: Adv Healthc Mater. 2023 Feb 25;12(11):2203237. doi: 10.1002/adhm.202203237 (PMC11468823; doi:10.1002/adhm.202203237)
Supplement: Supplementary file 1 — Supporting Information [file ADHM-12-2203237-s001.pdf]

ADVANCED HEALTHCARE MATERIALS

Supporting Information

for *Adv. Healthcare Mater.*, DOI 10.1002/adhm.202203237

Silk-in-Silk Nerve Guidance Conduits Enhance Regeneration in a Rat Sciatic Nerve Injury Model

*Lorenz Semmler, Aida Naghilou, Flavia Millesi, Sonja Wolf, Anda Mann, Sarah Stadlmayr, Sascha Mero, Leon Ploszczanski, Lisa Greutter, Adelheid Woehrer, Eva Placheta-Györi, Fritz Vollrath, Tamara Weiss and Christine Radtke**

Supporting Information:

Supplementary figures

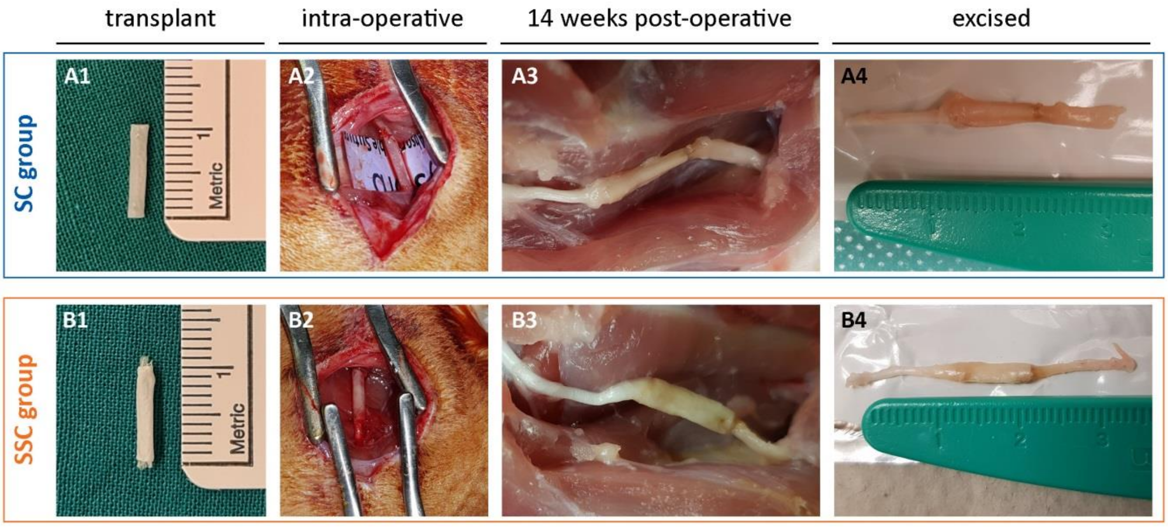

Supplementary Fig. 1: In vivo study overview. Representative images of the SC group **(A)** and SSC group **(B)**: silk fibroin conduit (A1) and silk-in-silk conduit (B1), after implantation (A2, B2), 14 weeks post-operative (A3, B3) and after isolation (A4, B4).

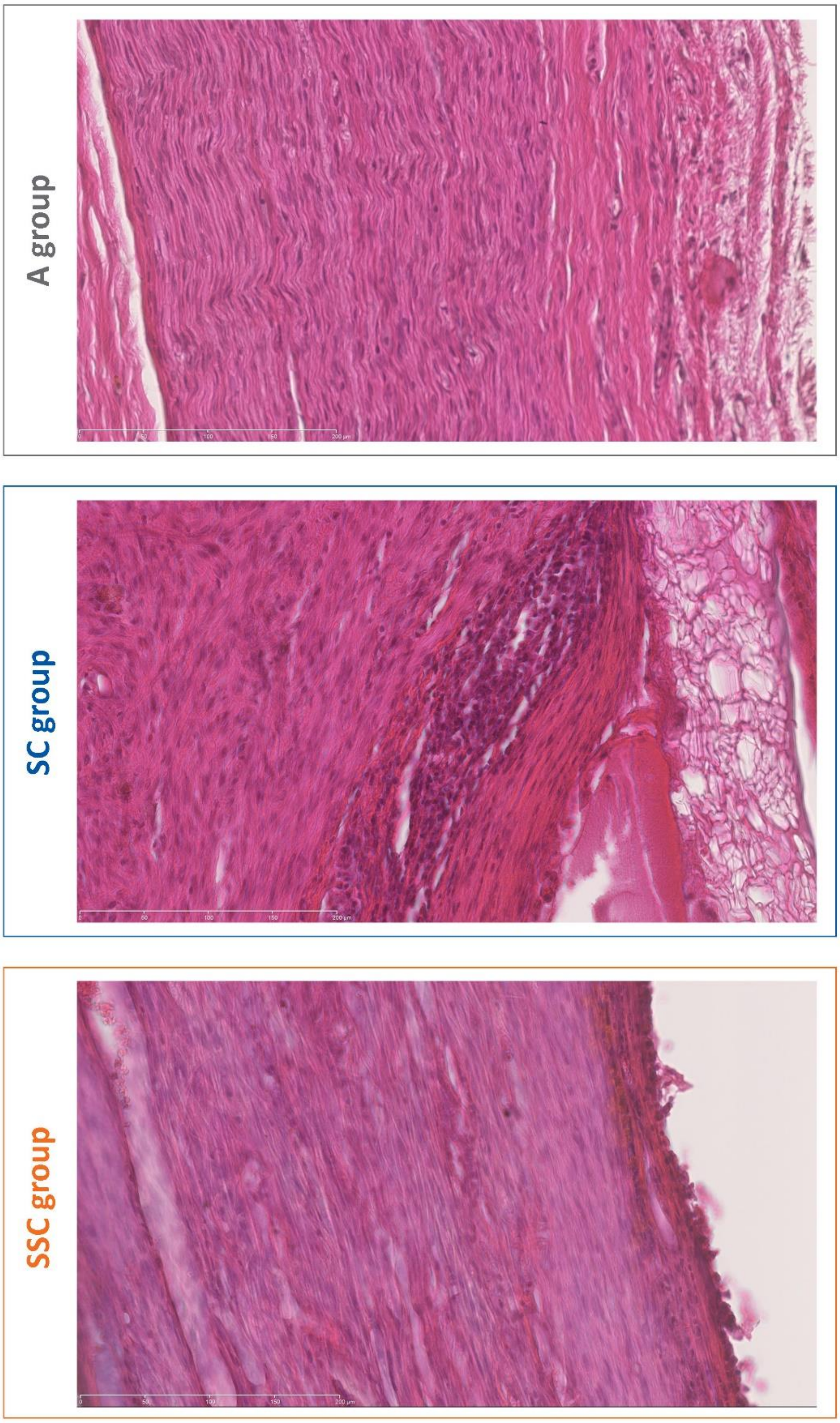

Supplementary Fig. 2: Hematoxylin and eosin staining of nerve sections of all groups 14 weeks post-surgery. Representative enlargements of hematoxylin and eosin stained nerve sections of the **(A)** autograft (A) group, **(B)** silk-tube (SC) group, and **(C)** silk-in-silk (SSC) group. Scale bars represent 200 µm.

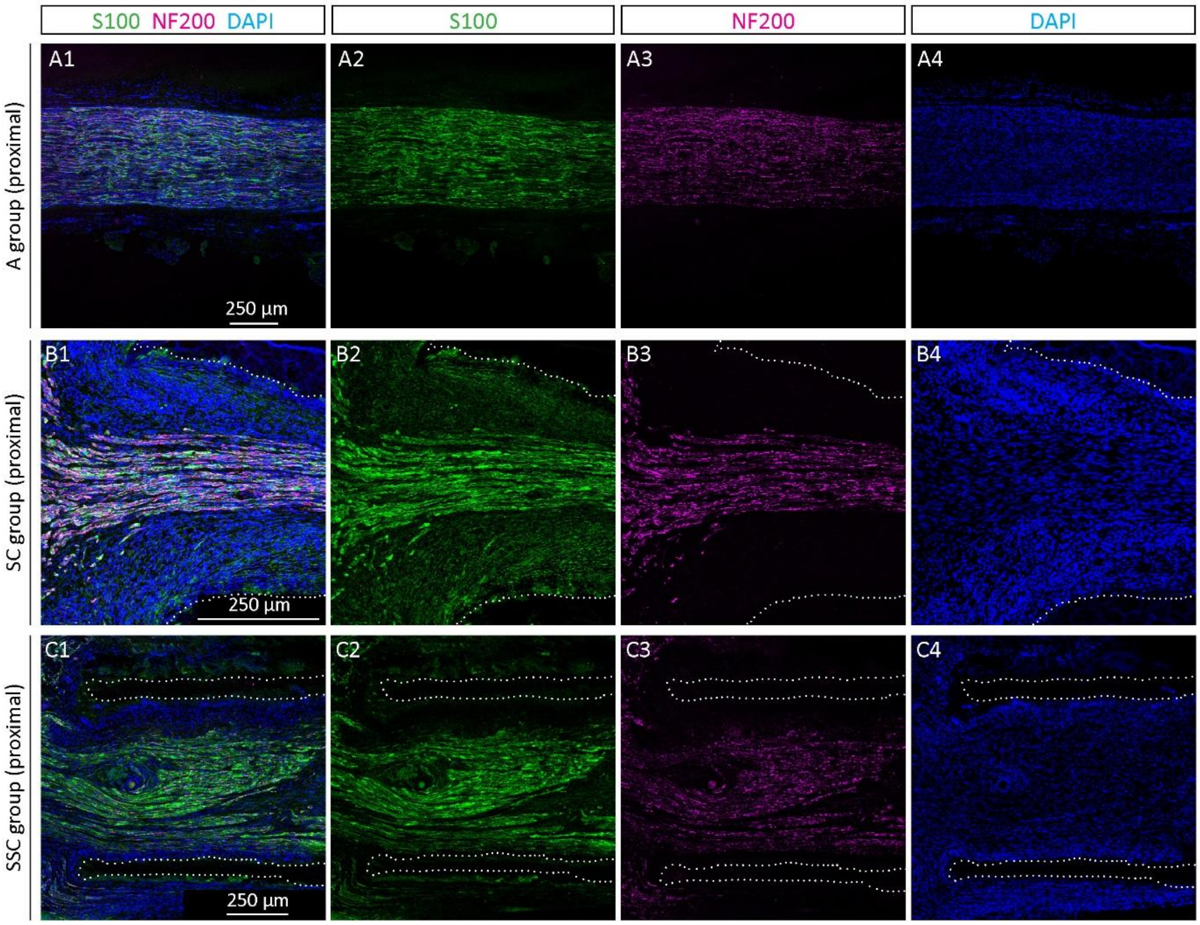

Supplementary Fig. 3: Immunostainings of longitudinal sections, proximal part, of all groups 14 weeks post-surgery. Representative immunofluorescence images of the proximal part of the **(A)** A group, **(B)** SC group, and **(C)** SSC group with merged channels (A1, B1, C1), S100 staining in green (A2, B2, C2), neurofilament 200 (NF200) staining in magenta (A3, B3, C3) and DAPI stain in blue (A4, B4, C4). The white dotted line indicates the silk conduit. In all groups, NF200 positive re-growing axons are with associated S100 positive Schwann cells.

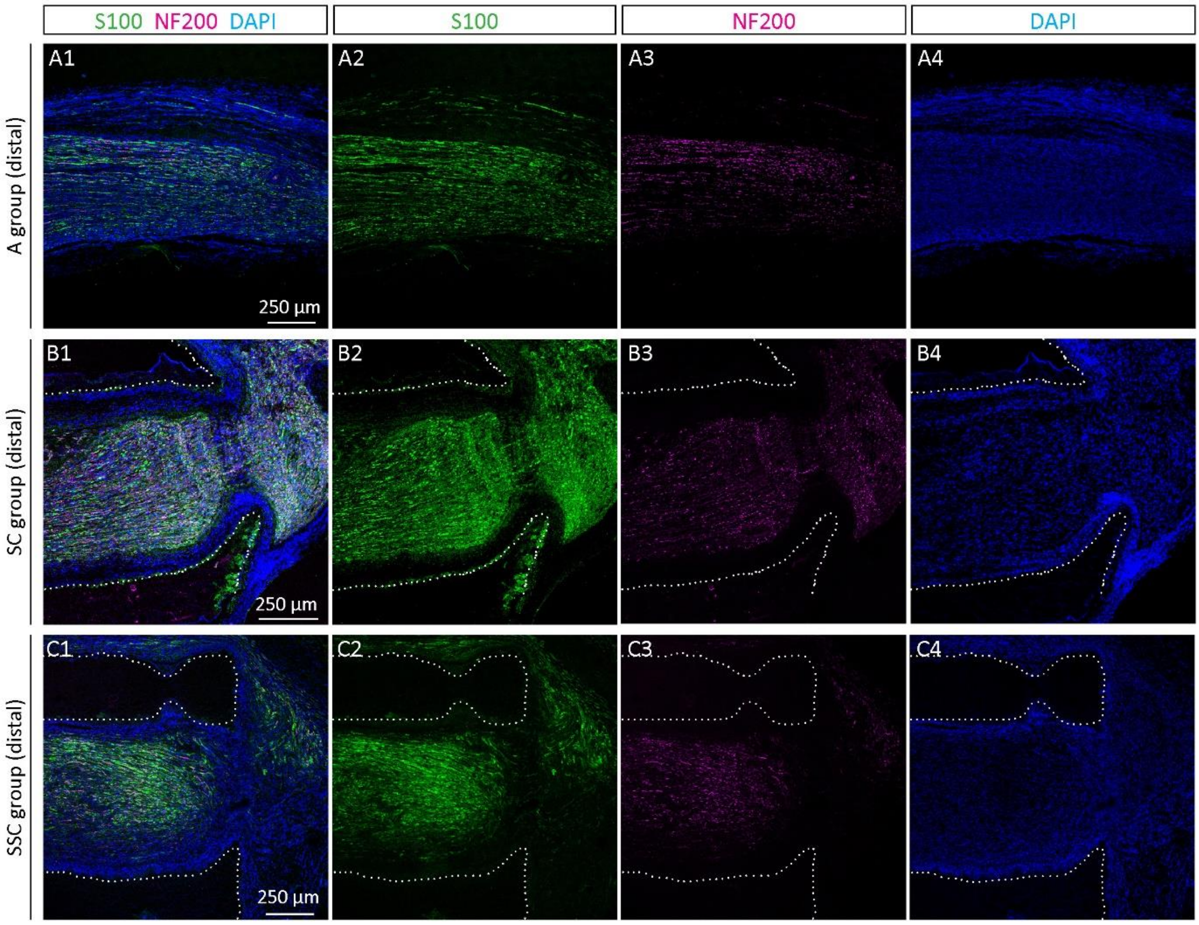

Supplementary Fig. 4: Immunostainings of longitudinal sections, distal part, of all groups 14 weeks post-surgery. Representative immunofluorescence images of the proximal part of the **(A)** A group, **(B)** SC group, and **(C)** SSC group with merged channels (A1, B1, C1), S100 staining in green (A2, B2, C2), neurofilament 200 (NF200) staining in magenta (A3, B3, C3) and DAPI stain in blue (A4, B4, C4). The white dotted line indicates the silk conduit. In all groups, NF200 positive re-growing axons are with associated S100 positive Schwann cells.
